# Collectanea Medica

**Published:** 1814-08

**Authors:** 


### COLLECTANEA MEDICA,

CONSISTING OF

### ANECDOTES, FACTS, EXTRACTS, ILLUSTRATIONS, QUERIES, SUGGESTIONS, &c.

RELATING TO THE

*History or the Art of Medicine, and the Auxiliary Sciences.*

---

*On the Use of Venesection in Diabetes Mellitus.* By JOSEPH AYRE, M.D. Physician to the Hull General Infirmary, and to the Penitentiary, and to the Lying-in Charity of Hull, &c. &c.

MR. C. of Castlethorpe, near Brigg, in Lincolnshire, farmer, aged 34, of laborious and temperate habits, and of robust form, was first seized with symptoms of diabetes mellitus, on the third day of a journey, in which he had undergone considerable bodily daily fatigue, and been exposed to much cold and rain, having taken, during that time, but a very few hours of natural repose. About the middle of April 1811, and six weeks after the attack, he applied to Mr. Rudkin, a respectable surgeon, of Brigg, and shortly after a physician of this place was consulted. From the commencement of the disease, the thirst had rapidly become urgent, and at this period was literally unquenchable, demanding, as ascertained by several trials, from 35 to 40 English pints of water, as the smallest quantity that could be dispensed with in the 24 hours. The urine was pale, and very sweet, corresponding, as it was conjectured, in quantity with the fluids taken, and voided almost involuntarily. The pulse small and quick; tongue white; skin very dry; appetite voracious; and bowels habitually and obstinately costive.

Immediately after consulting these gentlemen, he was put upon an animal diet, and directed to avoid all vegetable matters, and to take lime-water for his common drink. During the space of nine months, he strictly adhered to this regimen, and took the following medicines with the utmost regularity, viz. Peruvian bark, rhatany root, extract of logwood, alum, gum kino, sulphate of copper, tincture of cantharides, opium combined with sodorifics, the volatile and vegetable alkalies, the sulphuret of potass, and hepatized ammonia, but with only occasional and very trifling benefit. Towards the close of December, I was consulted, and found him labouring under the disease in its most aggravated form. His thirst was excessive, and such was its intensity towards evening, that his frequent calls for drink and micturition precluded all repose, until many hours after midnight. He was considerably reduced in flesh, and incapable of walking but a very short distance. His urine was highly saccharine, and in other respects indeed so altered, that it was his common practice to prefer it for rinsing his mouth, as the most effectual means he possessed for removing its clamminess. The voraciousness of the appetite, and the unperspirable state of the skin were unabated, and the bowels were still very costive. Sensible that a very full trial had been given to the ordinary remedies for this disease, and under the full assurances that nothing but death could result from their continuance, I determined to adopt a different plan, and having explained my views to the patient, and obtained his consent to the measure, I directed him to lose ℥xviii. of blood. This quantity, for reasons that need not be explained, was taken in two bleedings, viz. on the 6th and 10th of January, by Mr. Rudkin, with whom I corresponded, and to whom I am much indebted for the following reports, as well as for the obliging and intelligent manner with which he seconded my wishes.

1812. January 6th—13th.—After the first bleeding, the patient's pulse was increased from 80 to 96 in a minute, and is now 100, and weak. The symptoms of the disease are considerably abated; thirst not so excessive; the tongue, which has been white from the commencement of the complaint, is now but little so; urine less saccharine, particularly of a morning. The blood was of a dark colour, coagulating slowly, and the serum thick and adhesive, and of a milky appearance, but most so at the first bleeding. Lime water and animal diet continued, with an allowance of porter at the patient's request. The bowels kept regular by means of the oleum ricini.

13th—18th.—From the favourable change produced by the first bleeding, 16 ounces were taken yesterday. The blood was strongly marked with the buffy coat, but the pulse did not rise. The improvement, however, upon the whole is considerable.

18th—23d.—Since the last report, the thirst had increased, and the urine had become more abundant and saccharine; 16 ounces of blood were taken yesterday. The buffiness less considerable than at former bleedings, but the good effects are more considerable. Mr. C. feels his spirits and strength much improved, and thinks himself considerably better after this bleeding than after any of the former; the pulse is also stronger and less frequent, and the tongue clean.

23d—30th.—From the very considerable benefit derived from venesection, 20 ounces were taken on the 26th, which exhibited no buffiness on the surface. In the evening, feeling himself languid, he ate very heartily of fat meat, which appeared to disorder his stomach, and on the following day he complained of dizziness, and increased thirst. Urine sweet; pulse 110, and feeble; and tongue white. A bolus of calomel was given, which afforded relief, and the symptoms have gradually abated in violence.

January 30th—February 7th.—Has considerably improved since the last report. Yesterday 18 ounces of blood were taken, which was florid and free from siziness, though the serum was still milky; pulse 90, full, and stronger; has gained nine pounds in weight since he was first bled.

7th—13th.—For several days past has pursued his farming work; 16 ounces were taken yesterday, which was without buffiness, but the serum was still milky. Feels his spirits and strength greatly improved; sleeps well; thirst quite moderate, though the tongue is white, and the skin continues dry; urine occasionally saccharine, though not immoderate in quantity, having seldom occasion to rise more than once in the night.

13th—21st.—From an observation made by Dr. Watt, that an animal diet retarded the cure, a portion of vegetable food was begun with on the 16th. In the evening his thirst was increased, and the urine became sweet, with much prostration of strength. The night was restless, and the appetite was again voracious. These symptoms continued to increase with such violence, as to render a return to an animal diet indispensable: 18 ounces of blood were also taken, which was very sizy, but which had the immediate effect of removing the urgent symptoms. At one time, during the three days in which he used the vegetable diet, such was the intensity of his thirst, that he drank a gallon of water in 20 minutes.

21st—30th.—Since last report, nearly all the symptoms of diabetes have disappeared. The thirst and appetite are quite natural;

 urine urine in all respects natural; sleep quiet; pulse 86, and of good strength; spirits excellent; skin moist.

February 30th—March 27th.—Since last report there has been no return of the diabetic symptoms, and the general health and strength are so much improved, that Mr. C. goes about the ordinary business of his farm. After some laborious exertions on the 16th, he had a little increase of thirst; and, at his own request, 16 ounces of blood were taken on that day. Has gained seventeen pounds in weight since he was first bled.

March 27th—June 15th.—Since last report has had occasional returns of thirst, for which he was bled on the 2d of April, to the amount of 16 ounces, with instant relief, and 12 ounces were taken on the 9th, and the same quantity on the 30th of May, with the same result. Since that time, he has continued perfectly well, engaging in the most laborious employments of his farm, and rising at three or four every morning.

After the lapse of many months from the date of the last report, I had two or three times the satisfaction of seeing and learning from my patient, that he had continued perfectly well, and had acquired farther increase of weight. In the spring of this year, however, and about twelve months after his recovery, he came over to Hull for my advice, when I learnt that he had been labouring under a return of his disease for a few weeks. His complaint was now in many respects different from the former state of it. The common symptoms of diabetes mellitus were much less urgent, but the debility and languor were greater, and, what was still worse, there was a tightness in the chest, attended by dyspnœa and cough. Medicines were ordered for the pectoral symptoms, and 16 ounces of blood were taken, which afforded some little relief. Shortly after this, he was attacked by a disease of an erysipelatous nature, at that time prevailing, in which small blisters arose in different parts of his body, attended by fever, and followed by troublesome sores. I now saw him again, and found his pectoral symptoms much the same, but his debility much greater, and evidently increased by venesection, which had again been tried. It was at this time I learnt from him, with not less surprise than regret, that he had been in the practice, for many months past, of eating a portion of flour-pudding daily, and of making an unsparing use of potatoes. A strict adherence to an animal diet was recommended, but, under the very unfavourable circumstances then existing, it gave but little promise of benefit. In fact, the debility and other symptoms increased. The bold but successful practice employed in his former attack was now no longer admissible. The fatal crisis appeared fast approaching. During the succeeding fortnight he became gradually worse, and, towards the close of it, his dyspnœa was suddenly and alarmingly increased, so as to demand an attempt at relief by means of venesection. This however failed, and he shortly after became comatose, and in a few hours expired.—*Edinburgh Med. and Surg. Journal.*

### *Report of the Progress of the Sciences in France in 1813;* by J. C. Delametherie.

### *Animal Physiology.—On the Influence which the Temperature of the Air exercises in the Chemical Phænomena of Respiration.*

Respiration has been latterly regarded as a kind of combustion, viz. that of the carbon and hydrogen contained in the venous blood. The oxygen absorbed by this combustion forms carbonic acid and water.

It has been endeavoured to ascertain the quantity of atmospheric air which a man of a middling stature inspires at each inspiration, and expires at each expiration. It has been supposed that it was from 20 to 30 and even 40 cubic inches; but I have shown that this supposition is not correct. A man of a middling stature inspires only a few inches of atmospheric air. Now the atmospheric air contains only about one-fifth oxygen, or 0·21.

In the act of respiration a very small portion of this oxide inspired is combined with carbon, and forms carbonic acid.

Another portion of this oxygen is combined with a portion of inflammable gas, and there is a production or disengagement of water. But the greater part of this light portion of oxygen is not combined, and it is found in the air expired mixed with carbonic acid.

But Delaroche has discovered that there is a *production of azote.* He made a great number of experiments in order to determine the influence which the temperature of the air exercises in the chemical phenomenon of respiration. He placed at different temperatures animals in *manometres* or glass vessels with large apertures, hermetically closed by copper plates and screws. If we compare, he says, the results of the experiments made on one and the same animal placed in the same circumstances and at *different temperatures,* we shall see that almost in all the experiments upon the cold-blooded animals, the quantity of oxygen absorbed was a little greater when the temperature was low than when it was high.

The difference between the quantities of carbonic acid formed at different temperatures is still less considerable.

In all cases there is less carbonic acid produced than oxygen absorbed. *I concluded,* he says, *with M. Berthollet, that there was a production of azote.*

In an experiment made upon a hare, the manometre contained 0·7900 azote, 0·2100 oxygen.

After the experiment, the azote was 0·7991, the oxygen 0·1516, and the carbonic acid 0·0416.

There was therefore a production of 0·0091 of azote, and 0·054 of oxygen absorbed. I had remarked the same phenomena, and I have observed in my Essay upon pure Air, that in the air expired there was always a production of a portion of *impure air,* the azote of the new nomenclature.

Spallanzani has proved that a contrary effect takes place in the cold-blooded animals. My experiments, says M. Delaroche, prove also also that heat augments in a most remarkable manner the activity of respiration in these animals. The quantity of oxygen absorbed by frogs exposed to a heat of 27° has been in one experiment *double*, and in the other *quadruple*, to what it was when the external temperature did not exceed six or seven degrees.

*Animal Heat.*—Respiration being regarded as a kind of combustion, it has been considered as the principal cause of the heat of animals; but I have shown that too much stress has been laid on this cause.

1. *a.* We find that a man of middling stature only takes in at every inspiration a few cubic inches of atmospheric air. Now atmospheric air contains but little more than one-fifth of oxygen, or 0·21.

*b.* There is but a small quantity of this oxygen combined in respiration, certainly less than a cubic inch.

2. *a.* A man who sleeps tranquilly takes cold, although he breathes quite at his ease.

*b.* If he takes exercise, he acquires heat, and even perspires.

*c.* An animal exposed to a severe cold may perish if it does not take exercise. If, on the contrary, it moves or carries burdens, it preserves its life.

*d.* Consequently the muscular motion has the greatest influence on animal heat.

3. Oxygen gas contains very little heat; therefore the small portion which is combined in the act of respiration has produced very little heat.

I have concluded from these facts, that animal heat proceeded but in a very trifling degree from the caloric extricated from the oxygen inspired.

4. If animal heat proceeded from respiration, or from the combustion of the carbon in the act of respiration, the lungs ought to possess a greater degree of heat than the other parts of the system, as Brodie has asserted, which is not the fact. He thinks that animal heat is in a great measure under the influence of the nervous system and of the brain.

In the muscular movements the nervous system is in a state of activity more or less considerable; this is the reason that heat is produced in the animal body.

5. The fermentation of the various animal liquors contributes much to the heat of animals, for we know that every fermentable substance contracts heat. Now all the animal liquors are in a perpetual state of fermentation.

6. There are continual combinations in the animal economy which give new products, such as the phosphoric, uric, sebic, acids, glutine, fibrine, &c. &c. Now all these combinations are uniformly accompanied with an extrication of caloric.

7. The galvanic action is powerfully exerted among the various heterogeneous particles of the bodies of animals which ferment. This galvanism is very intense in the electric eel, &c. and contributes powerfully to animal heat.—*Phil. Mag.*

*Fatal*

### *Fatal Case of Hydrothorax, with Appearances on Dissection.* Communicated by Mr. A. ROBERTSON, Surgeon of H.M.S. Cydnus.

THE subject of this case was a man who had spent the greater part of his life in the active duties of a seaman, in almost every quarter of the globe,—a man certainly of an advanced age, (for 54 may be considered so, especially in a sailor,) yet remarkably squat, muscular, hale and strong for his years;—a man who would have been the last suspected to fall a martyr to hydropic affections; yet in him, "with all these appliances and means to boot," did the disease rapidly advance, and prove fatal on the *tenth* day from the very earliest feelings of indisposition!

He first made his appearance on the 17th of October 1813, complaining of an unusual sensation about the cartilago ensiformis, which he distinctly told me was not acute pain. He had first felt it at four o'clock that morning, on getting up to keep his watch. His pulse, tongue, and skin, as also (by his own account) his appetite and bowels, were perfectly natural. Thus no symptom of general or local illness being perceptible, he was sent away, with an injunction to call again, if he should feel himself worse.

He returned on the morning of the 20th October, and stated that the uneasiness in the lower part of the thorax had not left him, but that, on the contrary, it was now attended with slight cough and difficulty of respiration. His pulse and tongue were still natural; the temperature of his skin that of health. In fact, the only apparent symptom was cough, with some expectoration of a viscid tenacious pituita.

Under these circumstances, his bowels were opened, and kept free; diaphoretics also were prescribed, together with the emulsion usually employed in catarrhus benignus. By these he felt somewhat relieved.

No more prominent symptoms appeared till the 22d October. Heretofore his appetite had been good; his face expressive of health, and his tongue clean; but on that day the first was impaired, and the latter white; yet his pulse still preserved its natural tone and velocity. Difficulty of breathing and cough had increased, and the sensation at the scrobiculus cordis had become an oppressive sense of weight. From these particulars, I was led to suspect an incipient effusion of the serous sort, probably (as I then thought) into the bronchial tubes of the lungs: I therefore ordered the tinct. digitalis purp. in such quantity as the state of the stomach would admit; kept the bowels free; and soothed the cough by the same mixture as before.

His complaint seemed to be stationary till the 25th, when early in the morning he was seized with a severe fit of dyspnœa and coughing; his pulse was *now* quick, weak, and small; face pale and anxious; and he felt it impossible to lie in the horizontal posture. A drachm of æther nitros. administered at the time in a glass of peppermint water, gave him instant relief; but the former symptoms became, from that time, more and more violent.

The

The disease having now assumed so decided an attitude, the Tinct. digital. was increased, the spirit. ætheris nitros. repeated, when dyspnœa, more severe than ordinary, made it necessary; a large blister was applied to the sternum, and two grains of opium exhibited at night to procure sleep.

Neither at this nor at any time during the rapid progress of the disease was there any external anasarca, general or local; nor could fluctuation of fluid be felt in the chest, probably on account of the great firmness of the muscles, and the quantity of adipose substance accumulated over the ribs.

The above remedies were persevered in to the end. None gave him relief, excepting the draughts of æther, and cough-mixture; and the ease they procured him was very transient. He continued to become worse and worse: frequent retching was superinduced by the severity of the cough; his pulse was very quick and weak; debility gained ground rapidly; his stomach was so irritable that it rejected all medicines, and other ingesta but thin sago.

On the evening of the 27th of October, he was seized with a fit of dyspnœa; retching and cough more severe than any of the preceding; and expired suddenly during the paroxysm.

*Appearances on Dissection.*—On opening the body, I found that very strong adhesions were formed on both sides of the chest, betwixt the pleura costalis and pleura pulmonalis.

Both sacs of the pleura were prodigiously distended with a greenish serous fluid. The quantity was not ascertained by measurement, but I cannot possibly be deceived when I say it was *one gallon and a half!* In dissections of a similar nature, I do not remember to have seen such a profusion of water.

The pericardium contained, as nearly as I can judge, about a *pint* of fluid, of the same appearance with that in the cavity of the thorax. The heart itself was of an enormous size, at least *three times larger* than usual! The blood-vessels distributed on its muscular structure were whitish, and indurated, as if in a state of incipient ossification.

The lungs contained no tubercles, but were in every respect healthy.

The stomach was distended with flatus, but no marks of disease were to be traced on its coats.

The liver and other viscera had their natural appearance.—*Edin. Journ.*

---

### *On Sudden Death in Child-Bed, soon after Delivery.* By JOHN RAMSBOTHAM, M.D. Teacher of Midwifery.

THOUGH the act of *parturition* be not a morbid but a natural process, yet it is frequently, in the human female, attended in its progress with difficulty; and sometimes followed, after its completion, with fatality.

The disparity in the degree of danger and difficulty in the same process in woman and in the brute, so much in favour of the latter, is is not the effect of accident, but is the natural and necessary consequence of difference in form and structure. The erect position, produces also, in great part, the difficulties and dangers of human parturition. From the posture of the body—from the relatively larger size of the infantile head—from the structure and attachment of the placenta, requiring uterine contraction for its separation—from the changes which the abdominal contents and parts sympathizing with them, undergo upon the expulsion of the child and its appendages, a parturient woman is subjected to numerous hazardous incidents which arise during that process. These peculiar dangers are necessarily attached to and arise out of her form, and the specific nature of her condition.

It is true, that, generally, the natural powers do effect those changes during and after labour, which ensure the safety of the woman: but it is equally true, that, occasionally, the efforts of those powers are either suspended, or are so imperfectly exerted, as to leave the latter part of the process incomplete. In such instances, life is placed in a state of extreme hazard, until the necessary changes be completed.

My present intention is not to enter at large into the discussion of this most important subject: I shall therefore confine my observations to the danger every parturient woman has to encounter on the separation of the placenta; and on the sudden removal of pressure on the expulsion of the uterine contents: the latter, especially, is a source of danger, which, in my opinion, has not been sufficiently insisted upon, and practically attended to.

When the contractile efforts of the uterus have expelled the child, the placenta is commonly found lying in the vagina, separated from its uterine attachment, and may be removed, in proper time, without difficulty or danger. There is uniformly, on this separation or removal, a greater or less discharge of blood from those uterine vessels, which did communicate with the placenta, but which is checked by the contraction of the uterus; and in proportion to the degree of contraction, the chance of uterine hæmorrhage is increased or diminished. If, however, after the birth of the child, the placenta be not detached, uterine contractions of a slighter description are shortly resumed, and that mass is at first separated from, and afterwards expelled the uterine cavity. Now, if this natural process should not take place, and an increased discharge of blood ensue, the woman will incur a degree of danger commensurate with the rapidity of the hæmorrhage and the quantity of blood lost; and which danger will exist, till the placenta be ultimately removed, and the uterus firmly contracted.

Under a loss of blood from the uterine vessels, the patient experiences the same symptoms, as under violent or sudden loss of blood from any rupture or division of other blood-vessels. But, as the diameter of the uterine vessels, particularly of those communicating with the placenta, has considerably enlarged during the progress of pregnancy, and still remains much dilated, these vessels pour out their contents with a degree of velocity frequently inconsistent sistent with the order and course of the circulation: hence occasionally sudden death occurs, even before danger is apprehended.

Under the continuance of uterine hæmorrhage the woman becomes faint, which effect gradually increases with the loss of blood; the pulse is smaller and quicker; probably a state of complete syncope may ensue, during which the action of the heart is temporarily suspended or imperfectly exerted. In this state the hæmorrhage diminishes, or for the time entirely ceases. Yet, sometimes, after these symptoms have continued for a longer or shorter space, the patient becomes restless, sighs deeply and involuntarily, the pulse gradually loses its beat, and at length the circulation entirely ceases. But before the loss of sense, she complains of spasmodic constriction of the stomach, attended with violent pain.

The causes of such rapid and dangerous symptoms are obvious, viz. the depletion of the vascular system by the loss of that blood which is escaping from the uterine vessels. The hæmorrhage too is of a passive kind; the blood is flowing from these vessels, so long as the circulation is going on, for want of a power of collapsing in their extremities, or of diminution of their diameter by the contraction of the uterine parietes.

The usual progress of the symptoms attendant on unrestrained uterine hæmorrhage is nearly similar, whether the loss of blood take place before the removal of the placenta or afterwards. But though the contraction of the uterus be the desirable object in either case, yet some difference in treatment is required.

In hæmorrhage, threatening life before the removal of the placenta, especially if that mass be adherent to the uterine surface, and the natural powers seem inadequate to its separation, manual assistance is demanded. But, when hæmorrhage comes on after the placenta is brought away, such measures are to be had recourse to, and without loss of time, as appear most likely to produce a strong and permanent contraction of the uterus: this, and this alone, will restrain the flooding, and insure the woman's safety. To accomplish this end, pressure with the hand on the uterine tumour, and the external application of cold, must be ranked among the most conducive means.

Under some circumstances of uterine hæmorrhage, the exhibition of stimulants becomes absolutely necessary, to rouse the exhausted powers of vitality. When, for instance, in consequence of a rapid and great depletion, the action of the heart and arteries is nearly suspended, so that the pulse is almost imperceptible, and the woman seems just expiring, the immediate and free use of brandy, or any other stimulant, will usually restore the action of the sanguiferous system, renew those functions which appear nearly destroyed, and prove the means of saving life. But, when those objects are obtained, the further use of such means is not only unnecessary, but injurious.

I have offered these remarks on the effects of uterine hæmorrhage, for the purpose of contrasting the symptoms produced by loss of blood with those occasioned by another, and by no means an infrequent, cause of death after labour, viz. the removal of pressure from the parietes of the abdomen, and the contents of its cavity, as a consequence of the emptying of the uterus.

A woman sometimes appears safely put to bed after an easy and natural labour; she has suffered no unusual loss of blood on the separation and removal of the placenta; the uterus, on the application of the hand, is found well contracted, and the patient, thus far at least, promises to do well; but notwithstanding these favourable appearances, and perhaps even during the congratulations of her friends upon the termination of her sufferings, she complains of a degree of faintness, not indeed amounting to syncope, but attended with an inexpressible sensation of sinking; this is followed by restlessness, with an anxious depressed countenance, and occasionally by pain and a sense of constriction at the pit of the stomach; expressions of alarm in such words as these, "O! I shall die!" "I am dying!" are frequently repeated; by and bye the restlessness increases, the countenance becomes more dejected and ghastly, the pulse gradually fails in its stroke, the oppressive constriction on the epigastrium becomes intolerable, so as considerably to affect respiration; and, if relief to these symptoms be not speedy, she is shortly a corpse.

Such a termination of a natural process is as unexpected as it is sudden; it consequently strikes the attendants with astonishment and terror, and gives a shock to the feelings of those interested in the welfare of the patient, not to be described.

This unhappy occurrence has happened to women who have had a number of children, and who were moving in a respectable rank of life, in the enjoyment of every domestic comfort, and in possession of every necessary assistance. But I by no means intend to imply, that women who have had a number of children, or those who move in a superior sphere, are exclusively the subjects of this fatal termination of labour. I presume the situation of every lying-in woman renders her somewhat obnoxious to such an attack.

Although the above described case be as dangerous, and the result be as equally fatal as from uterine hæmorrhage, yet it differs in its nature and cause, and requires a distinct mode of treatment. I have before mentioned, that there is an obvious cause for the symptoms under uterine hæmorrhage, whereas in this case it is by no means evident; it can be surmised only, and is rather presumed than proved.

A very melancholy case of this description happened lately in London. A young lady was delivered of a first child after an easy labour; she appeared to go on very well for a short time, when she complained of a violent pain at the pit of the stomach, and soon after expired. A very accurate examination of the body was made, yet no satisfactory cause of death could be discovered.

What rational solution can be offered of such an unexpected and fatal event? and under circumstances too of promised security but a few a few minutes before it occurred? And, which is of far more importance, what means seem the most likely, on a similar attack, to avert the threatened danger?

It appears to me probable, that on the removal of pressure, by the sudden emptying of the uterus, the parietes of the abdomen and its contents do not readily accommodate themselves to the change thus induced; and, in consequence, some unfavourable impression is made upon the brain and nervous system, and thence transferred to the heart and circulation. An analogous event occasionally occurs after a great surgical operation; by which the system has sustained such a shock, as, under its effects, is incompatible with the continuance of life.

That a woman may die suddenly from the rupture of a vessel in the brain, or in the thoracic or abdominal cavities, during the violent effects of labour, is a conclusion sufficiently natural; but I suspect, in an accident of such rare occurrence, there would be symptoms of pressure on the sensorium in the one instance, and of internal hæmorrhage in the other; and the cause of death, on inspection, would be apparent. Besides, it would be the most likely to happen in lingering or difficult labours; whereas in the case now under consideration it takes place after the most easy, quick, and natural labours, wherein but little resistance to the passage of the child has been experienced, and is unattended with an unusual loss of blood: but our attention is principally excited by that alarming sense of sinking and peculiar and inexpressible anxiety. The progress of this unfortunate case must of necessity be rapid, and its termination sudden: the patient is either soon restored, or quickly lost; so that little time is allowed for reflection, and none for procuring additional assistance. The attack must also occur very soon after delivery; for if the necessary changes are accomplished—if the parts have had time to effect that relative accommodation which the change they have so lately undergone requires,—the woman is safe. Now and then a slight rigor succeeds delivery: though this is no dangerous symptom, yet it seems not improbable that it may be connected with the same cause.

The practical inference I would offer, as leading to a decided and successful mode of treatment of such cases, is this; to endeavour by the immediate and free use of stimulants to keep up the action of the nervous and sanguiferous systems, till the first changes in the circulation be effected, and till a mutual accommodation of the several parts within the abdomen has taken place. With this view, at the commencement of faintness *without loss of blood*, I would have recourse to the exhibition of brandy, or other spirit, undiluted or diluted according to the urgency of the symptoms and the rapidity of their progress, and in such quantity as may seem necessary to answer the intended purpose. That purpose being attained, and the patient relieved, further use of stimulants may do harm. The medicated stimuli, as æther, volatile spirit, cordial tinctures, &c. may of course be used with advantage. Moderate pressure upon the abdomen with the hand, or a bandage applied round the body, will assist the general intention; and the patient ought, on no consideration, to be allowed to raise herself from the recumbent posture, till she be so far recovered as to warrant security from the recurrence of the symptoms of alarm and danger.—*Apoth. Rep.*

